# France

**Published:** 1895-06

**Authors:** 


### LEGISLATION.

### New French Law Relative to the Preparation, Sale, and Distribution of Therapeutic Serums and Other Analogous Products.

The French Government, upon April 25, 1895, promulgated the following law :

Article 1. Attenuated viruses, therapeutic serums, modified toxins, and analagous products used for the prophylaxis and therapeutics of contagious diseases, and substances for injection of organic origin not defined chemically, applied to the treatment of acute and chronic affections shall not be either retailed gratuitously or for pay unless they have been in their manufacture and delivery subject to the authorization of the Government, given upon the advice of the consulting committee on public hygiene of France and of the Academy of Medicine.

These products will be approved by a temporary authorization which is revocable. They are subject to an inspection by a commission named by the proper Minister.

Art. 2. These products will be given to the public by pharmacists upon medical prescriptions. Each bottle or holder will bear the stamp of the place from which it comes and the date of its manufacture.

In urgent cases physicians are authorized to furnish these same products to their patients. When they are destined to be used gratuitously among the poor the bottles containing the products shall have on the glass the words, "public aid, gratuitous" (*Assistance publique—gratuit*). They can then be furnished outside of the shops and pharmacies, and under the surveillance of a physician in public establishments designated by the administration, which will have the liberty of procuring the products directly.

These conditions do not apply to the vaccine of Jenner, human or bovine.

Art. 3. The delivery of the substances mentioned in Article 1, by whatever name they may be called, will be assimilated to the sale and subject to the dispensations of Article 423 of the Penal Code, and to the law of the 27th day of March, 1871. Therefore, all those who shall have deceived concerning the nature of the substances which they know to be false and contaminated, and those who will have deceived or attempted to deceive concerning the quality of the substances delivered, will be punished by the penalties demanded in Article 423 of the Penal Code and by the Law of the 27th day of March, 1871.

ART. 4. All other infractions of the conditions of the present law will be punished by a fine of from sixteen to one thousand francs.

